# Fast and reliable production, purification and characterization of heat-stable, bifunctional enzyme chimeras

**DOI:** 10.1186/s13568-015-0122-7

**Published:** 2015-06-10

**Authors:** Mara Neddersen, Skander Elleuche

**Affiliations:** Institute for Technical Microbiology, Hamburg University of Technology, Kasernenstr. 12, 21073 Hamburg, Germany

**Keywords:** Bifunctional fusion enzyme, Cellulases, Extremophiles, Synergism, Thermozymes

## Abstract

Degradation of complex plant biomass demands a fine-regulated portfolio of glycoside hydrolases. The LE (*Lgu*I/*Eco*81I)-cloning approach was used to produce two enzyme chimeras CB and BC composed of an endoglucanase Cel5A (C) from the extreme thermophilic bacterium *Fervidobacterium gondwanense* and an archaeal β-glucosidase Bgl1 (B) derived from a hydrothermal spring metagenome. Recombinant chimeras and parental enzymes were produced in *Escherichia coli* and purified using a two-step affinity chromatography approach. Enzymatic properties revealed that both chimeras closely resemble the parental enzymes and physical mixtures, but Cel5A displayed lower temperature tolerance at 100°C when fused to Bgl1 independent of the conformational order. Moreover, the determination of enzymatic performances resulted in the detection of additive effects in case of BC fusion chimera. Kinetic measurements in combination with HPLC-mediated product analyses and site-directed mutation constructs indicated that Cel5A was strongly impaired when fused at the N-terminus, while activity was reduced to a slighter extend as C-terminal fusion partner. In contrast to these results, catalytic activity of Bgl1 at the N-terminus was improved 1.2-fold, effectively counteracting the slightly reduced activity of Cel5A by converting cellobiose into glucose. In addition, cellobiose exhibited inhibitory effects on Cel5A, resulting in a higher yield of cellobiose and glucose by application of an enzyme mixture (53.1%) compared to cellobiose produced from endoglucanase alone (10.9%). However, the overall release of cellobiose and glucose was even increased by catalytic action of BC (59.2%). These results indicate possible advantages of easily produced bifunctional fusion enzymes for the improved conversion of complex polysaccharide plant materials.

**Electronic supplementary material:**

The online version of this article (doi:10.1186/s13568-015-0122-7) contains supplementary material, which is available to authorized users.

## Introduction

As the major component of the plant cell wall, cellulose is the most abundant renewable biomass resource on earth. Due to the severe continuous depletion of crude oil and constant emission of greenhouse gases, effort has been taken to establish sustainable production of bioethanol from lignocellulose. In addition to efficient pretreatment methods to separate lignin, hemicellulose and cellulose, the efficient degradation of the latter polysaccharides into fermentable monosaccharide sugars, by the synergistic action of enzymes, is a bottleneck in lignocellulose conversion (da Costa Sousa et al. 2009; Hu et al. 2013). However, the economic commercialization of lignocellulosic biorefinery approaches is mainly hindered by the large costs to produce functional and stable biocatalysts for polysaccharide decomposition (Bornscheuer et al. 2014). The complex structure of lignocellulose is the major impediment to its degradation and requires the use of a portfolio of cellulases: endoglucanases (EC 3.2.1.4) randomly cleave internal β-1,4-glycosidic linkages, while cellobiohydrolases (EC 3.2.1.91) produce cellobiose by hydrolyzing chain ends of oligo- and polysaccharides, and finally, β-glucosidases (EC 3.2.1.21) produce glucose from cellobiose (Klippel and Antranikian 2011).

So far, most cellulases that exhibit enzymatic properties for industrial applications were isolated and characterized from wood-degrading fungi or mesophilic *Bacteria* (Kuhad et al. 2011). However, harsh industrial conditions certainly presume the exploitation of further enzyme sources. Moreover, conventional isolation and application techniques reached their limits in recent years, resulting in the development of versatile molecular biology techniques to engineer tailored biocatalysts (Bornscheuer et al. 2012). These candidates are being designed to overcome main drawbacks including limits in enzymatic specificity, efficiency and thermal instability.

The increasing demand for active biocatalysts capable of catalyzing the conversion of cellulose at elevated temperatures or in the presence of solvents allows the reasonable application of enzymes that are isolated from extremophilic microorganisms, so called extremozymes (Elleuche et al. 2014). Such *Bacteria* and *Archaea* thrive in the harshest places on earth like hot springs, sea ice, solfataric fields and the deep sea and represent a treasure chest of industrially applicable biocatalysts encoded in their genomes. Due to cultivation limits, metagenomic analyses have greatly facilitated the identification of cellulases and additional biocatalysts from extremophiles (Chow et al. 2012; Graham et al. 2011; Schröder et al. 2014).

Suitable candidates with comparable biochemical properties can be used for fine-regulated processes to efficiently degrade plant-material for the production of monosaccharides. To increase coupled catalytic action of single enzymes, several strategies were tested including enzyme cocktails, artificial cellulosomes and fusion enzymes (Bülow et al. 1985; Elleuche 2015; Morais et al. 2012; Resch et al. 2013; Rizk et al. 2012). In this context, end-to-end gene fusion has been proven to be a competent method for the construction of lignocellulose degrading bi- and multifunctional enzymes (Adlakha et al. 2012; Fan et al. 2009a; Hong et al. 2007; Kang et al. 2015; Lee et al. 2011). Using this method, a polypeptide is capable of catalyzing two or more distinct reactions. Thus, the number of enzymes that have to be produced will be minimized.

In a previous study, a highly active endoglucanase, Cel5A, from the thermophilic anaerobic bacterial species *Fervidobacterium gondwanense* DSM 13020 was shown to tolerate its fusion to another protein either at its N- or C-terminus without loosing its catalytic ability to degrade β-1,4-linked cellulosic materials (Rizk et al. 2015). In this study, *F. gondwanense* Cel5A and a β-glucosidase from a hydrothermal spring metagenome exhibiting comparable heat-stable and heat-active properties were fused in both orientations. Detailed characterization of fusion constructs and equal mixtures of parental enzymes showed that the close proximity is advantageous. However, improved performance was only detectable in one orientation that is superior over the other.

## Materials and methods

### Strains and culture conditions

*Escherichia coli* strain NovaBlue Singles™ (Merck KGaA, Darmstadt, Germany) was used for plasmid propagation and maintenance and *E. coli* M15[pREP4] (Qiagen, Hilden, Germany) was the host for heterologous expression of cellulase-encoding genes and for production of bifunctional fusion proteins. Antibiotics ampicillin (100 µg/ml) and kanamycin (50 µg/ml) was supplemented to Luria Bertani (LB) for selection of plasmids. Protein production took place in a 1.2-l fed-batch fermentation culture at 37°C in medium prepared as described elsewhere (Horn et al. 1996). Gene expression was induced by the addition of 1.0 mM isopropyl-β-d-1-thiogalactopyranoside (IPTG) when an optical density OD_600_ = 25–30 was reached. After 4 h of incubation at a constant temperature of 37°C, cells were harvested by centrifugation resulting in an average wet weight of 80–90 g. Cell pellets were frozen at −80°C for storage and further used for purification approaches.

### Site-specific cloning of cellulase-encoding genes into recipient expression vector

To express cellulase-encoding genes as HIS-ENZYME-STREP variants in *E. coli*, the endoglucanase gene *cel5A* (JQ972696) from *F. gondwanense* and the archaeal β-glucosidase gene *bgl1* (HG326254) from an Azorean hydrothermal spring metagenome were amplified by polymerase chain reaction (PCR) and flanked with *Lgu*I and *Eco*81I restriction sites. Cloning strategy to produce plasmid pQE-30-LE::*cel5A* by ligation of *cel5A* into expression vector pQE-30-LE has been described as proof-of-principle for LE-(*Lgu*I/*Eco*81I) cloning technique in a previous project (Marquardt et al. 2014). In a parallel approach, *bgl1* was amplified with primers Bgl1-LguI-f (5′-CAGCTCTTCCTCAGTAAAGTTCCCTAAAGGGTTCATG, *Lgu*I-restriction site is underlined) and Bgl1-Eco81I-r (5′-GTCCTGAGGAAGTAAGAACGTTTGGAAATTTACTGTATTC, *Eco*81I-site is underlined) using pQE-80L::*bgl1* as template (Schröder et al. 2014). Afterwards, PCR amplicons were subcloned into pJET1-2 (Life Technologies, Darmstadt, Germany). After sequence verification and *Lgu*I/*Eco*81I double digestion, genes were ligated into *Lgu*I-linearized pQE-30-LE to give plasmid pQE-30-LE::*bgl1* (Eurofins, Ebersberg, Germany). To produce bifunctional fusion constructs, plasmid pQE-30-LE::*cel5A* was either hydrolyzed with *Lgu*I or *Eco*81I, and mixed with *Lgu*I/*Eco*81I double digested *bgl1* fragment to give plasmids pQE-LE::BC and pQE-LE::CB, respectively (Table 1).

**Table 1:** Plasmids used in this study

Plasmid	Characteristics	References
pQE-30-LE	HIS-STREP, separated by *Lgu*I/*Eco*81I overlapping restriction site	Marquardt et al. (2014)
pQE-30-LE::*cel5A*	HIS-Cel5A-STREP	Marquardt et al. (2014)
pQE-80L::*bgl1*	HIS-Bgl1	Schröder et al. (2014)
pQE-30-LE::*bgl1*	HIS-Bgl1-STREP	This study
pQE-30-LE::CB	HIS-Cel5A-Bgl1-STREP	This study
pQE-30-LE::BC	HIS-Bgl1-Cel5A-STREP	This study
pQE-30-LE::C^E294A^B	HIS-Cel5A^E294A^-Bgl1-STREP, inactivated endoglucanase	This study
pQE-30-LE::BC^E294A^	HIS-Bgl1-Cel5A^E294A^-STREP, inactivated endoglucanase	This study
pQE-80L::*bgl1*_G395	HIS-Bgl1^E395G^, inactivated β-glucosidase	Schröder et al. (2014)
pQE-30-LE::CB^E395G^	HIS-Cel5A-Bgl1^E395G^-STREP, inactivated β-glucosidase	This study
pQE-30-LE::B^E395G^C	HIS-Bgl1^E395G^-Cel5A-STREP, inactivated β-glucosidase	This study

### Site-directed mutagenesis of fusion genes

Two different strategies were used to generate point mutation variants of *cel5A* and *bgl1*, respectively. An inverse PCR (iPCR) was done to introduce E294A substitution into the endoglucanase-encoding gene using primers Cel-294(a-c)-F (5′-CAGTTTTTCTTGGAGCATTCGGTGCTTATTC, mutated nucleotide is underlined, E294A) and Cel-294(a-c)-R (5′-GAACATTGTTCTTCTTTGCCCAGTCGCTTAC, reverse primer for iPCR). A parallel inactivation of a recognition site for enzyme *Eco*RI (GAATTC was changed to GCATTC) was used to screen for positive clones after plasmid isolation (Rizk et al. 2015). Mutation of endoglucanase-encoding gene in 5′-position to *bgl1* resulted in plasmid pQE-30-LE::C^E294A^B, while a mutated endoglucanase-gene located at the 3′-end led to the production of plasmid pQE-30-LE::BC^E294A^ (Table 1). Inactivation of β-glucosidase was achieved by a substitution of E395G (Schröder et al. 2014). A gene encoding an inactivated variant was amplified from plasmid pQE-80L::*bgl1*_G395. Primers Bgl1-LguI-f and Bgl1-Eco81I-r were used in PCR to create *bgl1*^E395G^ with flanking *Lgu*I and *Eco*81I restriction sites. The fragment was subcloned into pJET1.2 (Life Technologies, Darmstadt, Germany) and sequenced (Eurofins, Ebersberg, Germany). After digestion with *Lgu*I and *Eco*81I, the gene *bgl1*^E395G^ was either ligated into *Lgu*I pre-digested pQE-30-LE::*cel5A* to give plasmid pQE-30-LE::B^E395G^C or into *Eco*81I linearized pQE-30-LE::*cel5A* to give pQE-30-LE::CB^E395G^ (Table 1).

### SDS-PAGE and western blot and zymograms

A 12% sodium dodecyl sulphate-polyacrylamide gel electrophoresis (SDS-PAGE) was applied to separate proteins produced in *E. coli*. Visualization was achieved either using Coomassie Brilliant Blue staining or Pierce® Silver Stain Kit (Life Technologies, Darmstadt, Germany). The tool “Compute pI/Mw” from ExPASy Proteomics Server (http://www.expasy.org) was used to calculate molecular weights of proteins for their identification on SDS-PAGEs. A semidry western blotting system was used to transfer proteins from SDS-PAGE to Roti®-PVDF membrane (Roth, Dautphetal-Buchenau, Germany) and a combination of His-Tag® Monoclonal Antibody/Goat Anti-mouse FgG AP conjugate or Strep-Tag® II Monoclonal Antibody/Goat Anti-mouse FgG AP conjugate enabled detection of tagged proteins on the membrane with the His-tag® AP Western Reagents Kit (Merck KGaA, Darmstadt, Germany). In-gel activity assays (Zymograms) were done using carboxymethyl-cellulose (CMC) to measure activity of endoglucanase (i) and esculin for β-glucosidase (ii). Moreover, side activity (β-galactosidase) of the latter enzyme was determined with 5-bromo-4-chloro-3-indolyl-β-d-galactopyranoside (X-gal) (iii). (i) In case of endoglucanase, a thin layer of 2% (w/v) agar–agar containing 0.1% (w/v) CMC in 10 mM Tris-HCl, pH 7.5 was prepared. Samples were heated at 70°C for 5 min or 98°C for 10 min and run on SDS-PAGE. Denaturating agents were washed out in 2 × 30 min in 10 mM NaPO_4_, pH 7.0 mixed with 25% (v/v) isopropanol, followed by two washing steps for 10 min in 10 mM NaPO_4_, pH 7.0. Afterwards, the gel was incubated on top of the 0.1% (w/v) CMC-containing agar layer and incubated for 90 min at 70°C. CMC-agar was stained for 20 min using 0.1% (v/v) congo-red and washed for 5 min using 1 M NaCl to detect endoglucanase activity as halos. (ii) In case of β-glucosidase-activity, samples were prepared according to (i) and separated on a SDS-PAGE followed by a 1 min washing step in A. dest, 60 min washing in 1% (v/v) Triton X-100 and another step for 1 min in A. dest. Afterwards, the gel was incubated with 0.1% (w/v) esculin hydrate and 0.01% (w/v) ammonium ferric citrate in 10 mM NaPO_4_, pH 7.0 for 60 min at 70°C. (iii) To detect activity towards X-gal, samples were prepared and washed with A. dest and Triton X-100 according to (ii) and incubated for 60 min at 70°C in 10 mM NaPO_4_, pH 7.0 containing 0.3 mg (w/v) X-gal and 1% (v/v) dimethylformamide.

### Purification of recombinant proteins

To purify HIS-STREP double-tagged proteins, an amount of 5 g of frozen cell pellet from fed-batch fermentation was solubilised in 25 ml lysis buffer (50 mM NaH_2_PO_4_, pH 8.0, 300 mM NaCl, 10 mM imidazole, The QIAexpressionist, Qiagen, Hilden, Germany) and passed three times through French press (2000 psi, Spin Aminco, Spectronic Instruments, Leeds, UK) for complete cell lysis. Cell debris was precipitated by centrifugation and soluble proteins were collected in the supernatant. A heat-precipitation step (70°C, 10 min) followed by centrifugation was applied to separate denatured, heat-labile proteins. Afterwards, heat-stable proteins in the supernatant were loaded onto polypropylene columns equipped with Ni-NTA agarose (both Qiagen, Hilden, Germany) for purification of HIS-tagged proteins using washing (50 mM NaH_2_PO_4_, pH 8.0, 300 mM NaCl, 20 mM imidazole, The QIAexpressionist, Qiagen, Hilden, Germany) and elution buffer (50 mM NaH_2_PO_4_, pH 8.0, 300 mM NaCl, 300 mM imidazole, The QIAexpressionist, Qiagen, Hilden, Germany). Active fractions were pooled and dialysed against buffer W (100 mM Tris-HCl, pH 8.0, 150 mM NaCl, 1 mM EDTA, IBA—Solutions for Life Sciences, Göttingen, Germany) for STREP-tag affinity chromatography (Schmidt and Skerra 2007). Subsequently, Ni-NTA purified proteins were loaded onto polypropylene columns (Qiagen) filled with *Strep*-Tactin Superflow® (IBA,—Solutions for Life Sciences Göttingen, Germany). After washing, bound proteins were eluted using buffer E (100 mM Tris-HCl, pH 8.0, 150 mM NaCl, 1 mM EDTA, 2.5 mM desthiobiotin, IBA—Solutions for Life Sciences, Göttingen, Germany) and active fractions were pooled for further analysis.

### Protein activity assays

Plate assays were done by growing *E. coli* M15[pREP4] expressing single or fusion genes on LB-medium supplemented with ampicillin, kanamycin and 0.1 mM IPTG. Cells were grown over night at 37°C, before overlaying with AZCL-dye coupled HE-cellulose (Megazyme, Bray, Ireland) containing agarose for identification of endoglucanase-activity or 1.2% screening-agarose (50 mM sodium acetate, 2.5 mM CaCl_2_ × 2 H_2_O, 170 mM NaCl, 2.5 mM esculin hydrate, 0.4 mM ammonium ferric citrate) for β-glucosidase activity, respectively, and incubated at 70°C. Protein concentrations were determined according to the assay developed by Bradford (1976). Enzymatic activity towards cellulosic polysaccharides was quantified spectrophotometrically at 546 nm using the 3,5-dinitrosalicylic (DNS) acid-assay (Bailey 1988). The release of reducing sugar ends was measured using the carbohydrate substrate β-glucan (barley, low viscosity, Megazyme, Bray, Ireland) as model substrate. A standard reaction sample was composed of a mixture containing enzyme and 0.5% (w/v) β-glucan incubated in 10 mM NaPO_4_ buffer, pH 7.0. Reaction took place in 10 min at 80°C. The amount of enzyme needed to catalyze the release of 1.0 µmol of reduced sugar ends per minute was defined as one unit. Enzymatic activity of β-glucosidase was measured towards 2 mM of 4-nitrophenyl-β-d-glucopyranoside (4-NP-β-d-GP) in 10 mM NaPO_4_ buffer, pH 7.0 by incubation for 10 min at 80°C under optimal conditions. Subsequently, activity assay was stopped by addition of 10 mM Na_2_CO_3_ and incubation on ice. The optical density was determined at OD_410_. One unit of catalytic activity was defined as the amount of enzyme needed to release 1 µmol 4-nitrophenol per min under optimal conditions. To investigate proper reaction conditions, experiments were carried out between 20 and 100°C and pH 2.0 and 11.0. Heat stability tests were done by incubation of enzyme samples in 10 mM NaPO_4_ buffer, pH 7.0 at different temperatures between 64 and 90°C in a TGradient cycler (Biometra, Göttingen, Germany). Afterwards, samples were cooled down on ice, before they were applied in standard measurements.

High performance liquid chromatography (HPLC) was used to determine oligosaccharides, cellobiose and glucose resulting from β-glucan and cellobiose degradation by cellulases. A 1260 Infinity LC system equipped with Hi-Plex Na and Hi-Plex H columns and with a R_i_-detector from Agilent was applied with MilliQ-water used as mobile phase at a flow rate of 0.3 ml/min (Hi-Plex Na column) and 0.6 ml/min (Hi-Plex H column), respectively (Agilent, Waldbronn, Germany).

## Results

### Generation of bifunctional fusion enzymes

The aim of the study was to genetically link a gene (*cel5A*) encoding a glycoside hydrolase family 5 endoglucanase from extreme thermophilic bacterium *F. gondwanense* to an open reading frame (*bgl1*) encoding a glycoside hydrolase family 1 β-glucosidase from a hydrothermal spring metagenome (Klippel 2011; Schröder et al. 2014). To express single and fusion genes under identical conditions, open reading frames were ligated into vector pQE-30-LE harbouring HIS-and STREP-tag encoding sequences separated by a sequence for a -Gly-Ser-Ser-Ser-Gly- linker derived from an overlapping LE-restriction site (*Lgu*I and *Eco*81I) (Marquardt et al. 2014). A cloning approach using restriction endonucleases *Lgu*I and/or *Eco*81I enabled a step-by-step continuous ligation of two fragments in vector pQE-30-LE. Restriction/ligation of acceptor plasmid pQE-30-LE::*cel5A* led to the creation of *cel5A*-*bgl1* and *bgl1*-*cel5A* fusions. Expression of genes resulted in the production of proteins flanked with a HIS-tag at the N-terminus and a STREP-tag at the C-terminus. Linker amino acid residues were -Gly-Ser-Ser-Ser- between HIS-tag and N-terminal moiety and -Ser-Ser-Gly- between C-terminus of the target protein and STREP-tag. Two proteins in a fusion construct were separated by an additional -Ser-Ser- linker peptide.

**Figure 1:**
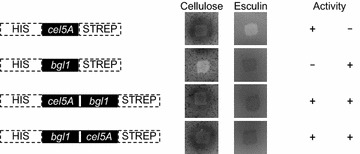
Qualitative analyses of enzymatic activity towards endoglucanase- and β-glucosidase-specific substrates. Graphic illustrations indicate the structure of single and fusion proteins, while plate assays display catalytic activity of mono- and bifunctional cellulases. AZCL-HE-cellulose and esculin were used as respective substrates for endoglucanase, and β-glucosidase.

Expression of cloned genes was tested on plate-assays using AZCL-HE-cellulose containing plates for endoglucanase activity and esculin-containing plates to detect β-glucosidase activity. *E. coli* M15[pREP4] harbouring plasmid pQE-30-LE::*cel5A* displayed endoglucanase-specific activity, while maintenance of plasmid pQE-30-LE::*bgl1* resulted in activity towards esculin. As expected, expression of both fusion constructs from plasmids pQE-30-LE::CB and pQE-30-LE::BC led to the hydrolysis of AZCL-HE-cellulose and esculin, respectively (Figure 1). Mutation of both catalytic glutamate residues, E294A in Cel5A and E395G in Bgl1, revealed functionality of each enzyme as fusion partner in both orientation variants (Additional file 1: Figure S1).

### Production and purification of enzymes

Expression strain *E. coli* M15[pREP4] harbouring plasmid derivatives of pQE-30-LE encoding for single or fusion genes were grown in high-cell density fermentation and crude extracts were prepared for protein isolation. Two purification steps using double-tag properties enabled the separation of fusion proteins including the ability to control the separation from degradation products containing single tags. Production and purification of single constructs HIS-Cel5A-STREP and HIS-Bgl1-STREP displayed the potential of two-step affinity chromatography of proteins that were produced with the pQE-30-LE system in *E. coli*. Both proteins were produced in recombinant form and purified in small scale (Additional file 1: Figure S2). The double-tagged variant of Cel5A was purified to apparent homogeneity (Additional file 1: Figure S2A), while a purification approach using Bgl1 led to the detection of an additional, smaller HIS-tag protein (Additional file 1: Figure S2B). It might be possible that a proteolysed, truncated HIS-tagged Bgl1 variant without STREP-tag interacts with full-length Bgl1 and therefore remained at low amount in the eluate after *Strep*-Tactin Superflow® purification. The recent observation that Bgl1 forms a tetramer under native conditions supports this hypothesis (Schröder et al. 2014).

**Figure 2:**
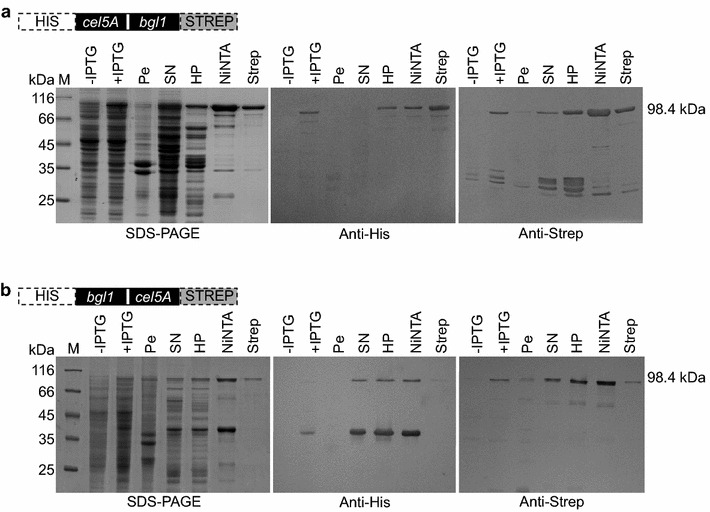
Purification of double-tagged fusion constructs produced in *E. coli*. **a** Purification of Cel5A-Bgl1 fusion construct. SDS-PAGE analysis and western blot of crude cell extracts and purification steps. **b** Visualization of Bgl1-Cel5A fusion protein by SDS-PAGE and western blotting analyses. *M* molecular weight marker, −*IPTG* total cellular protein, no induction, +*IPTG* crude extracts, 1 mM IPTG, *Pe* pellet fraction, insoluble cell debris, *SN* supernatant, soluble proteins, *HP* heat precipitation, heat stable proteins, *NiNTA* affinity chromatography step 1, *Strep* affinity chromatography step 2. Molecular weights are indicated aside.

To produce endoglucanase/β-glucosidase fusion constructs, *E. coli* M15[pREP4] was transformed either with plasmid pQE-30-LE::CB or pQE-30-LE::BC, respectively. SDS-PAGE and western blotting analyses of crude extracts and samples from purification steps revealed the identification of fusion proteins of an approximate mass of 100 kDa, which is in good agreement with the predicted molecular weights (98.4 kDa) of the full-length proteins (HIS-tag—1.3 kDa, STREP-tag—1.1 kDa, Cel5A—38.7 kDa, Bgl1—57.3 kDa). Interestingly, impurities exhibiting STREP-tagged degradation products were observed when Bgl1 was produced as C-terminal fusion partner (Figure 2a), while HIS-tagged truncated protein variants remained in NiNTA-purified fractions in fusion proteins with Bgl1 being at the N-terminus (Figure 2b). However, in case of HIS-Cel5A-Bgl1-STREP purification, STREP-tagged degradation products are not completely washed out during affinity chromatography using NiNTA-agarose. These results indicate that truncated Bgl1-STREP constructs probably interact with HIS-Cel5A-Bgl1-STREP proteins, thereby even remaining in the final eluate (Figure 2a). Moreover, a HIS-Bgl1 degradation construct is detectable in NiNTA-eluate when crude extract from HIS-Bgl1-Cel5A-STREP producing cells is applied onto NiNTA-agarose containing column (Figure 2b). Utilization of an additional STREP-tag purification step did not result in complete separation of the fusion protein from the degradation product, probably again due to Bgl1-multimerization (data not shown). Therefore, NiNTA-purified samples were heated up to 70°C for 10 min prior to application on *Strep*-Tactin Superflow® column resulting in efficient purification of HIS-Bgl1-Cel5A-STREP (Figure 2b).

**Figure 3:**
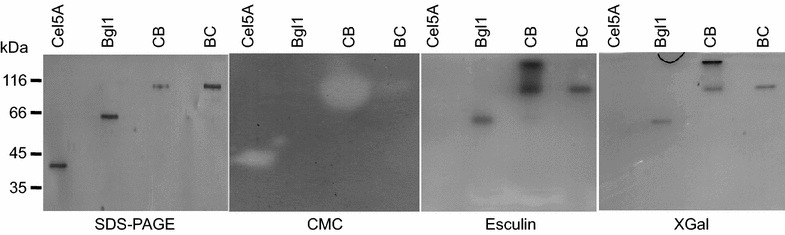
Zymograms to analyze catalytic activity of fusion enzymes. SDS-PAGE of purified single and fusion enzymes were loaded after denaturation (incubation at 70°C for 5 min in SDS-containing buffer). Protein bands were detected with silver staining. In case of in-gel activity assays, enzymes were washed with Triton X-100 and incubated at optimal conditions in reaction buffer. Utilization of soluble CMC-cellulose was used to detect the activity of endoglucanase, while esculin and X-Gal were applied to visualize the catalytic performance of β-glucosidase.

Zymograms using substrates for endoglucanase (CMC) and β-glucosidase (Esculin) were applied to investigate functionality of purified constructs (Figure 3). Activity towards CMC clearly indicates the functionality of endoglucanase, when produced as single protein and in both orientations as fusion enzyme. Fusion constructs and β-glucosidase alone displayed activity towards esculin and side-activity towards X-Gal, indicating functionality and the capability to be renaturated in SDS-PAGE. However, catalytic activity was detected only after sample heating at 70°C for 5 min prior to gel loading, while heating at 98°C for 10 min in sample buffer resulted in complete inactivity and loss of refolding ability. As a control experiment, purified β-glucosidase was loaded on a SDS-PAGE after heating at 70 or 98°C, respectively and used for zymogram assay, indicating that tertiary conformation is impaired by heat, resulting in non-reversal refolding. Incubation for 5 min at 70°C already resulted in two distinct bands on SDS-PAGE, indicating the presence of two forms of the enzyme (denaturated and native monomer). A signal at 59.9 kDa indicates complete denaturation and is also found after incubation at 98°C, while the protein seems to migrate faster through SDS-PAGE when the tertiary structure is partially retained at 70°C. Moreover, only the folded enzyme form displayed activity in a zymogram (Additional file 1: Figure S3).

### Catalytic properties of bifunctional cellulases

**Figure 4:**
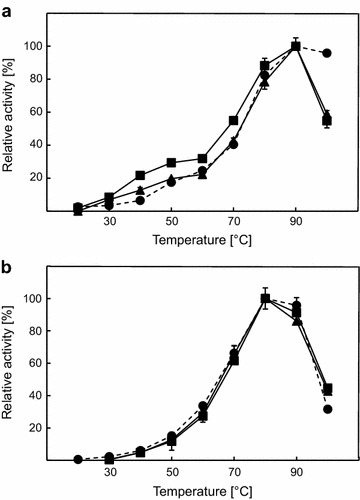
Influence of temperature on fusion and single constructs. **a** Purified proteins were tested using β-glucan as substrate at different temperatures and constant of pH 6. **b** Measurement of enzymatic activity towards 4-NP-β-d-GP. Experiments were done using Cel5A (*filled diamonds*, *dashed line*), Bgl1 (*filled circles*, *dashed line*), CB (*filled squares*, *continuous line*) and BC (*filled triangles*, *continuous line*). All experiments were done in triplicate.

**Figure 5:**
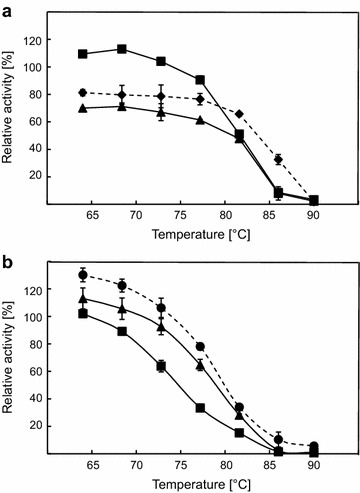
Effect of temperature on stability of fusion constructs. **a** Protein activity tests using β-glucan as substrate. **b** Enzymatic tests with 4-NP-β-d-GP. Proteins were incubated in 10 mM NaPO_4_ buffer, pH 7.0 for 60 min at different temperatures (64.0, 64.0, 68.4, 72.8, 77.2, 81.6, 86.0 and 90.0°C) in a gradient PCR-cycler. Experiments were done using Cel5A (*filled diamonds*, *dashed line*), Bgl1 (*filled circles*, *dashed line*), CB (*filled squares*, *continuous line*) and BC (*filled triangles*, *continuous line*). A control experiment with protein pre-incubated at 22°C was set to 100%.

**Figure 6:**
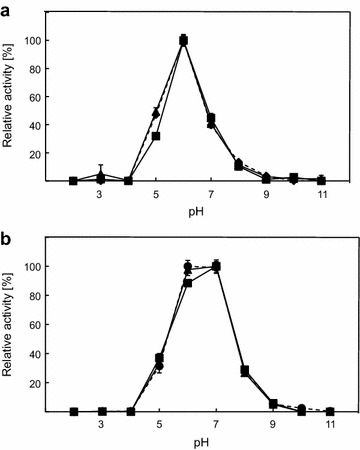
Influence of pH on catalytic activity of fusion constructs compared to single proteins. **a** Experiments were done with β-glucan as substrate at different pH and constant temperature of 80°C. **b** Enzymatic activity of β-glucosidases using 4-NP-β-d-GP as substrate. Experiments were done using Cel5A (*filled diamonds*, *dashed line*), Bgl1 (*filled circles*, *dashed line*), CB (*filled squares*, *continuous line*) and BC (*filled triangles*, *continuous line*). All experiments were done in triplicate.

Catalytic activity as a function of temperature and pH was measured using β-glucan and 4-NP-β-d-GP as substrates for endoglucanase and β-glucosidase. The activity was measured between 20 and 100°C with fusion constructs displaying optimal activity at 90°C for β-glucan and 80°C for 4-NP-β-d-GP. However, both fusion constructs displayed decreased activity towards β-glucan at 100°C when compared to Cel5A alone (Figure 4a). Due to the fact that inactive β-glucosidase fused to endoglucanase in constructs CB^E395G^ and B^E395G^C displayed the same result, this effect might be due to fusion mediated conformational changes of the enzyme (Additional file 1: Figure S4B). To prove this, singular Cel5A was mixed with singular Bgl1 and the activity profile according to temperature was compared to Cel5A alone (Additional file 1: Figure S5). Temperature profiles of Cel5A in mixture and alone were highly comparable proving that faster inactivation of tandem constructs is due to artificial fusion. Activity towards 4-NP-β-d-GP was not influenced by fusion of β-glucosidase to endoglucanase, when compared to enzymatic performance of Bgl1 (Figure 4b). Control experiments using fusion constructs with inactivated endoglucanase displayed identical results (Additional file 1: Figure S4A). Thermostability tests indicated that Cel5A was more stable as N-terminal fusion partner at low temperatures (up to 77.2°C), while both fusion constructs were instable compared to Cel5A alone when incubated at 81.6 and 86°C for 60 min. Incubation at temperatures between 60 and 70°C even resulted in thermoactivation for CB fusion construct (Figure 5a). Comparable activation effects were also obtained for CB and Cel5A after incubation for 10 min at lower temperatures (data not shown). Complete inactivity was observed for all constructs after incubation at 90°C for 60 min. In contrast to these results, activity of Bgl1 was decreased in fusion constructs. However, Bgl1 was also activated at temperatures between 64 and 68.4°C when fused at the N-terminus, but the single enzyme was more active at all conditions tested compared to fusion proteins (Figure 5b). Linking proteins did not influence the catalytic behaviour at all when different pH values were tested. Cel5A is optimally active at a pH of 6.0 and retains more than 40% of activity between pH 5.0 and 7.0. Almost identical results were measured with fusion constructs CB and BC, respectively (Figure 6a). Bgl1 and both fusion constructs retained more than 20% of catalytic activity towards 4-NP-β-d-GP between pH 5.0 and pH 8.0. The optimal pH was shown to be between 6.0 and 7.0 (Figure 6b).

### Enzyme kinetics and HPLC-analyses revealed additive effects of cellulase activities

Kinetic parameters were investigated using β-glucan as substrate for endoglucanase Cel5A and 4-NP-β-d-GP for β-glucosidase Bgl1 (Table 2). Substrate affinities for single constructs were determined to be K_M_ = 0.12% using β-glucan for Cel5A and K_M_ = 0.41 mM using 4-NP-β-d-GP for Bgl1, respectively. Affinity of enzymes in fusion construct BC was only slightly shifted (0.13% and 0.53 mM), while combined activity of both enzymes in construct CB displayed a lowered affinity towards β-glucan (KM = 0.23%). To accurately compare specific activities of fusion and single constructs, catalytic activity of Bgl1 in fusion constructs was adjusted to molecular weights of the single protein partner in the fusion enzymes. For example, His-tagged Bgl1 accounts for 49.2% of total molecular weight (98.4 kDa) in fusion construct CB, indicating that v_max_ = 744.0 U per mg of total fusion protein can be adjusted to 1,256.7 U per mg of Bgl1 as fusion partner in CB. Therefore, C-terminal fusion of Bgl1 resulted in a reduced activity towards 4-NP-β-d-GP (61.8% residual activity) compared to Bgl1 alone, while activity level of N-terminal Bgl1 is increased 1.2-fold. It is not possible to calculate the catalytic activity of Cel5A in fusion constructs using β-glucan in DNS-assays, because Bgl1 converts released cellobiose to glucose, which also exhibits reducing ends that are also measured. However, combined activity of both enzymes towards β-glucan was reduced compared to Cel5A (1,816.9 U/mg) alone, indicating that the catalytic performance of the endoglucanase was impaired by fusion at the N-terminus (822.6 U/mg) and C-terminus (1,205.9 U/mg). To get a deeper insight into enzymatic performance of Cel5A in fusion enzymes, site-directed mutagenesis was applied to inactivate Bgl1 moieties. Constructs CB^E395G^ and B^E395G^C enabled the detection of endoglucanase activity alone. As expected, catalytic activity of Cel5A in mutated constructs was reduced to 191.1 U/mg and to 536.1 U/mg, respectively, while the substrate affinity was only slightly affected, proving a parallel release of reducing sugar ends by Bgl1 in double-active fusion enzymes (Table 2).

**Table 2:** Kinetic parameters of biomass degrading enzymes

Constructs	Substrate, β-Glucan		Substrate, 4-NP-β-d-GP	
	K_M_ (%)	v_max_ (U/mg)	K_M_ (mM)	v_max_ (U/mg)
Cel5A	0.12	1,816.9	–	–
Bgl1	–	–	0.41	2,031.2
CB	0.23	822.6	0.53	744.0 (1,256.7)^a^
BC	0.13	1,205.9	0.53	1,472.3 (2,470.3)^a^
CB^E395G^	0.25	77.2 (191.1)^b^	–	–
B^E395G^C	0.18	216.6 (536.1)^b^	–	–

^a^Catalytic activity of Bgl1 calculated under consideration of single enzyme’s molecular weight in fusion construct.

^b^Catalytic activity of Cel5A calculated under consideration of single enzyme’s molecular weight in fusion construct.

**Figure 7:**
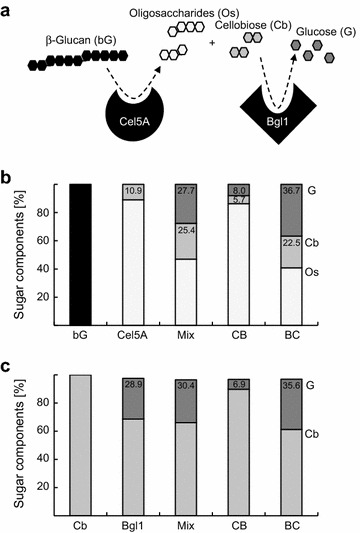
HPLC-analyses to investigate β-glucan degradation. **a** Schematic diagram depicting endoglucanase (Cel5A)-mediated degradation of β-glucan (bG) to give oligosaccharides (Os) and cellobiose (Cb). The β-glucosidase Bgl1 converts cellobiose to glucose (Glu). **b** Quantitative detection of released sugar components from 0.5% (w/v): β-glucan degradation by Cel5A, mixture of Cel5A and Bgl1 (Mix) and fusion constructs. Enzymes were used in 1:1 ratio in all assays. Concentration of fusion constructs was determined based on molecular weight of Cel5A moiety. **c** Quantitative detection of released glucose from 0.5% (w/v) cellobiose degradation by Bgl1, mixture of Cel5A and Bgl1 (Mix) and fusion constructs. Enzymes were used in 1:1 ratio in all assays. Concentration of fusion constructs was determined based on molecular weight of Bgl1 moiety. *Colour code* of sugar components in **a**, **b** and **c**: bG *black*, Os *light grey*, Cb *grey*, Glu *dark grey*.

To investigate positive effects in construct BC and inactivation effects in CB in more detail, HPLC-analyses were conducted. Barley β-glucan contains 32% β-1,3-linkages in addition to β-1,4-linkages, which are not hydrolyzed by endoglucanase resulting in the accumulation of various sized oligosaccharides with cellobiose being the lowest weight sugar compound released (Additional file 1: Figure S6). Utilization of different stationary phases to distinguish between small oligosaccharides, cellobiose and glucose enabled the detection of combined activities of Cel5A and Bgl1. The endoglucanase releases oligosaccharides and cellobiose from β-glucan and the latter is further processed by Bgl1 to give glucose (Figure 7a). Endoglucanase Cel5A produced oligosaccharides and 10.9% cellobiose from β-glucan when incubated for 24 h, while more then 50% of oligosaccharides were converted to cellobiose and glucose (27.7% glucose + 25.4% cellobiose) in the presence of Bgl1 mixed with Cel5A indicating potential product inhibition of Cel5A alone (Figure 7b). In contrast to these results, utilization of the BC fusion enzyme resulted in the determination of ~60% of di- and monosaccharides (36.7% glucose + 22.5% cellobiose) from β-glucan. Due to the decreased activities of the Cel5A and Bgl1 moieties in CB (see also v_max_, Table 2), fused Bgl1 only converted little amounts of cellobiose to glucose (13.7% release of glucose and cellobiose). The slight increase in cellobiose and glucose by CB compared to Cel5A alone might also be a result of product inhibition effects. This is also in good agreement with the observation that around 50–60% of totally released cellobiose is converted to glucose when both enzyme activities are present (Mixture: 51.6% glucose from 100% cellobiose, CB: 57.6%, and BC: 62.0%) (Figure 7b).

Furthermore, Bgl1A released almost comparable amounts of glucose from cellobiose when incubated as single enzyme (28.9% glucose from cellobiose), in mixture with Cel5A (30.4%) and in fusion protein BC (35.6%). The catalytic activity of Bgl1 in CB is decreased, which is also in good agreement with determined enzyme kinetics (Figure 7c, Additional file 1: Figure S7; Table 2). Moreover, comparable amounts of Bgl1 activity (each in mixture, CB and BC) were measured by final glucose yields in tests using either cellobiose as substrate or β-glucan (Figure 7b, c), indicating that no channelling effects were observed, but intermediate products were taken from the reaction mixture.

## Discussion

A consortium of cellulases and hemicellulases is mandatory for the efficient degradation of complex lignocellulosic plant materials (Bornscheuer et al. 2014; Khandeparker and Numan 2008; Rizk et al. 2012). Cost effective production of such enzymes is crucial for the sustainability of lignocellulosic bioethanol. Therefore, the generation of artificial enzyme chimeras is a promising approach to improve the cost-efficient degradation of plant-derived biomass. Different strategies are applied to produce multifunctional enzyme systems including enzyme cocktails, cellulosomes or xylanosomes and hybrid fusion enzymes (Conrado et al. 2008; Hu et al. 2013). However, all these different techniques without simple mixtures of singular enzymes are limited by the immense size of linked partners. There are two main strategies to circumvent the problem of high molecular weights: (1) enzymes can be truncated or even reduced to their catalytic site, or (2) proteins are chosen that do not contain domains in addition to the catalytic region. Cellulases are often modular multi-domain enzymes composed of carbohydrate-binding modules or predicted domains of unknown function (Bergquist et al. 1999). However, the thermozymes used in this study are compact enzymes that do only contain catalytic domains allowing the production of limited sized bifunctional fusion enzymes (Klippel 2011; Schröder et al. 2014).

The LE-cloning strategy has been especially designed to allow the continuous and easy integration of fusion partners in different orders into a growing vector system. Each clone (product and entry) can be used as a new entry-clone (Marquardt et al. 2014). The proper arrangement of fusion enzymes on one polypeptide has been shown to be important for efficient catalytic activity and was even shown to be advantageous for improved thermostability (Hong et al. 2007; Lee et al. 2011). In fact, extensive characterization of fusion constructs in comparison with parental enzymes is mandatory to understand their functional equivalency (Fan et al. 2009b). The fusion of the endoglucanase from *F. gondwanense* downstream of the β-glucosidase from an Azorean hot spring metagenome displayed catalytic properties that were not influenced by fusion of the two polypeptides with regard to pH range. However, activity of endoglucanase was reduced at 100°C compared to the single Cel5A and enzyme mixtures, which was probably a result of modified protein conformation. Structural data would be important to shed some light on fusion-mediated conformational changes influencing performance of endoglucanase and β-glucosidase. In contrast to this result, the β-glucosidase maintained the temperature optimum and activity range of the parental enzyme, when fused either to the N- or C-terminus of the endoglucanase. However, the specific catalytic activity of Bgl1 was reduced by fusion to the C-terminus, but slightly increased when produced as the N-terminal fusion partner enzyme. Shifts in pH and temperature optima by fusion of protein candidates were also described in other examples (Fan et al. 2009b; Ribeiro et al. 2011; Zhao et al. 2013).

The most interesting part in enzyme fusions is the possible improvement of catalytic properties including synergism, increased product yield and product channelling. In our case, BC was superior over the opposite orientation, resulting in increased total activity and improved thermostability of Bgl1. In contrast to these results, complete catalytic inactivity of a β-glucosidase (BglB) as a result of genetic fusion has been shown by the generation of a bifunctional β-glucosidase/endoglucanase from *Thermotoga maritima*. BglB was completely inactive when fused as the N-terminal partner, while both enzymes exhibited hydrolyzing activity in the opposite direction. However, fusion resulted in 70% reduced activity even in the orientation with BglB downstream of endoglucanase Cel5C (Hong et al. 2007). Lower or higher specific activities of fusion proteins were often reported, but there are only rare descriptions of bifunctional enzymes that were superior over the free enzymes because of true synergistic and conformational effects (Adlakha et al. 2012; Hong et al. 2007; Riedel and Bronnenmeier 1998; Rizk et al. 2015). BC released around 9% more glucose from β-glucan than the enzyme mixture, which was probably caused by the increased catalytic activity of β-glucosidase and not by intermediate channelling. In agreement with this hypothesis, activity of Bgl1 towards cellobiose was also slightly increased when fused to the N-terminus compared to the singular enzyme. Kinetic parameters were determined to shed more light on these effects.

The K_M_ of β-glucosidase was determined to be 0.4–0.5 mM for single enzyme and fusions, which is in the same range as observed for Bgl1 produced with a N-terminal HIS-tag without C-terminal tag in a previous study (Schröder et al. 2014). The determination of kinetic parameters using fusion enzymes CB and BC with the substrate β-glucan is difficult because of combined enzyme activities on initial substrate and intermediate products (Figure 7a). However, both fusion constructs displayed reduced activity towards β-glucan when compared to parental Cel5A, indicating that fusion negatively influenced the performance of the endoglucanase. It is thus hard to compare kinetics of Cel5A in fusion enzymes with the parental enzyme in both orientations. Therefore, mutation constructs containing inactivated β-glucosidase were generated to determine catalytic activity of endoglucanase without additional release of glucose from cellobiose mediated by Bgl1. The outcome displayed that activity of endoglucanase is significantly reduced in both enzyme chimeras. Interestingly, HPLC analyses revealed that the final product yield (glucose and cellobiose) of fusion enzyme BC is improved compared to 1:1 enzyme mixtures of identical molar concentrations. This result indicates that a secondary effect is important in addition to conformation-mediated activity reduction of Cel5A. Product analyses using HPLC were done after 24 h of incubation compared to 10 min activity assays used to determine enzyme kinetics probably indicating that the thermostability of fusion enzymes compared to single enzymes must be also taken into account when incubation took place at 60°C (Figure 5). Finally, such an effect is most probably influenced by the release of cellobiose as well, which inhibits the catalytic activity of endoglucanase.

Fusion of genes to produce multifunctional enzymes is an interesting tool for industrial application, due to improved catalytic activity as well as lower production costs from minimized numbers of recombinant polypeptides. The main objective is to create artificial chimeric enzymes that are superior over monofunctional biocatalysts in the hydrolysis of natural substrates. Generation of bifunctional fusion enzymes in both orientations was shown to be important in random fusion studies due to opposite increasing and reducing activity effects on identical partners. Moreover, the LE-cloning system can allow the incorporation of additional fusion partners into the established vector system to easily screen for superior multifunctional fusion enzymes containing additional cellobiohydrolases, cellulose binding modules or hemicelluloses in the future. However, structural determinations are highly recommended to understand conformational effects and to use the BC-construct for rational design studies to produce novel multifunctional biocatalysts.

## Additional files

**Additional file 1:** **Figure S1.** Schemes and plate assays of point mutation constructs. **Figure S2.** Expression of double-tagged cel5A and bgl1 genes in E. coli. **Figure S3.** SDS-PAGE analysis and zymogram of Bgl1 after incubation at two different temperatures. **Figure S4.** Influence of temperature on mutated fusion constructs compared to singular Cel5A and Bgl1. **Figure S5.** Influence of temperature on singular Cel5A and Cel5A in enzyme mixture. **Figure S6.** HPLC analyses to investigate catalytic activity of fusion enzymes towards cellobiose. **Figure S7.** HPLC analyses to investigate catalytic activity of fusion enzymes towards β-glucan.

## Abbreviations

4-NP-β-d-GP: 4-nitrophenyl-β-d-glucopyranoside
BC: Bgl1/Cel5A
CB: Cel5A/Bgl1
CMC: carboxymethyl-cellulose
DNS: 3,5-dinitrosalicylic
HPLC: High performance liquid chromatography
iPCR: inverse polymerase chain reaction
IPTG: isopropyl-β-d-1-thiogalactopyranoside
LB: Luria Bertani
PCR: polymerase chain reaction
X-gal: 5-bromo-4-chloro-3-indolyl-β-d-galactopyranoside
