# Supplementary material for: Fast and reliable production, purification and characterization of heat-stable, bifunctional enzyme chimeras
Source: AMB Express. 2015 Jun 10;5:33. doi: 10.1186/s13568-015-0122-7 (PMC4460186; doi:10.1186/s13568-015-0122-7)
Supplement: Supplementary file 1 — Additional file 1: Figure S1. Schemes and plate assays of point mutation constructs. Figure S2. Expression of double-tagged cel5A and bgl1 genes in E. coli. Figure S3. SDS-PAGE analysis and zymogram of Bgl1 after incubation at two different temperatures. Figure S4. Influence of temperature on mutated fusion constructs compared to singular Cel5A and Bgl1. Figure S5. Influence of temperature on singular Cel5A and Cel5A in enzyme mixture. Figure S6. HPLC analyses to investigate catalytic activity of fusion enzymes towards cellobiose. Figure S7. HPLC analyses to investigate catalytic activity of fusion enzymes towards β-glucan. [file 13568_2015_122_MOESM1_ESM.pdf]

AMB Express

Supplementary Material

Fast and reliable production, purification and characterization of heat-stable, bifunctional enzyme chimeras

Mara Neddersen, Skander Elleuche*

Hamburg University of Technology, Institute for Technical Microbiology, Kasernenstr. 12, D-21073 Hamburg, Germany

*Correspondence to Skander Elleuche

e-mail: skander.elleuche@tuhh.de

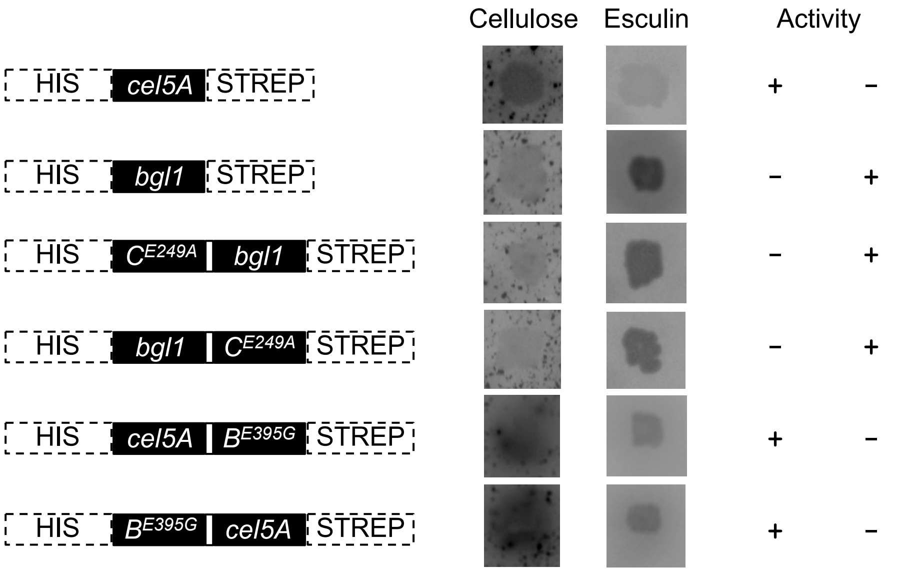

Fig. S1 - Schemes and plate assays of point mutation constructs. Structure of single and fusion proteins is indicated aside the plate assays. AZCL-HE-cellulose and esculin were used to investigate activity of endoglucanase and β-glucosidase, respectively.

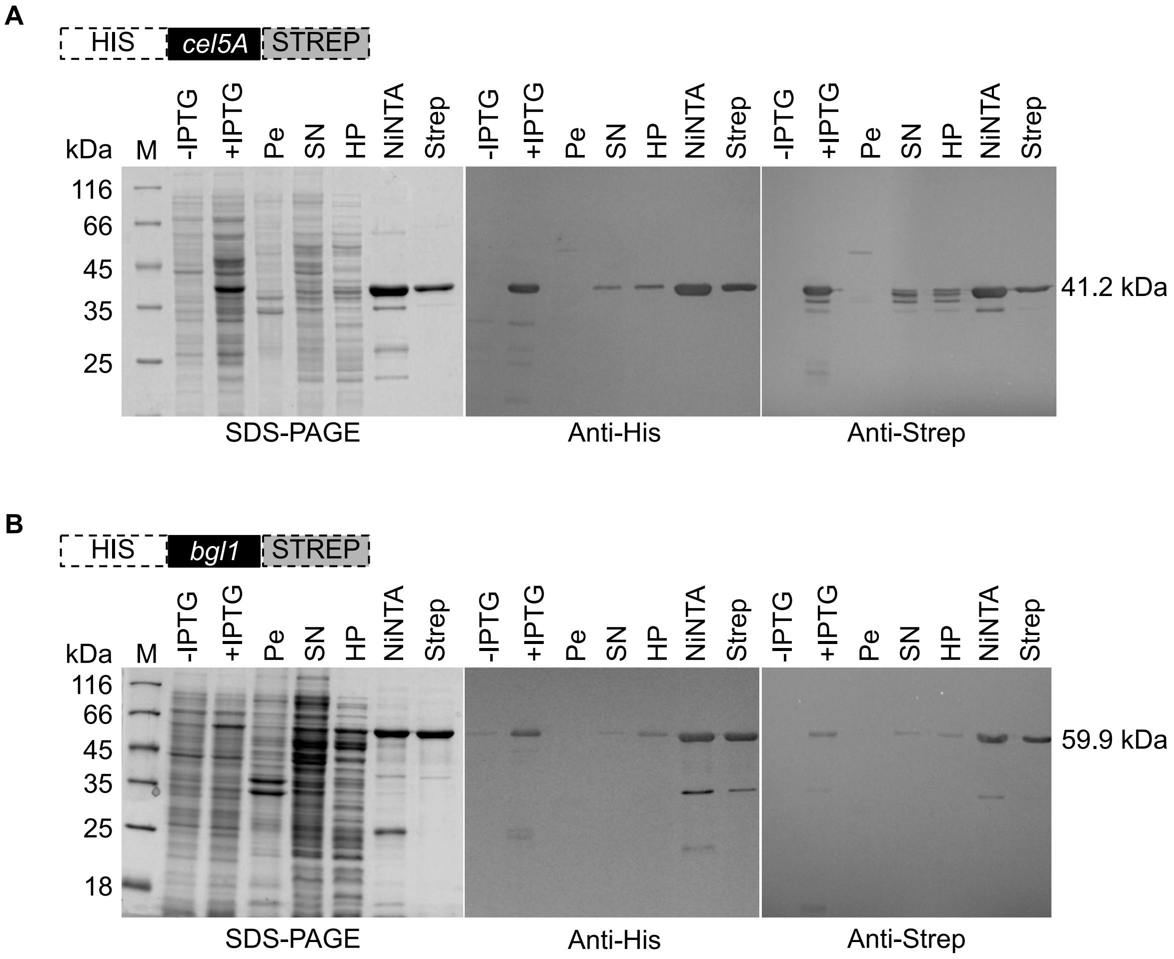

Fig. S2 - Expression of double-tagged *cel5A* and *bgl1* genes in *E. coli*. (A) SDS-PAGE and Western blot to analyze purification steps of double-tagged Cel5A produced in *E. coli* (B) Purification of Bgl1. Abbreviations are as follows: M – Molecular weight marker, -IPTG – total cellular protein, no induction, +IPTG – crude extracts, 1 mM IPTG, Pe – Pellet fraction, insoluble cell debris, SN – supernatant, soluble proteins, HP – heat precipitation, heat stable proteins, NiNTA – affinity chromatography step 1, Strep – affinity chromatography step 2. Molecular weights are indicated aside.

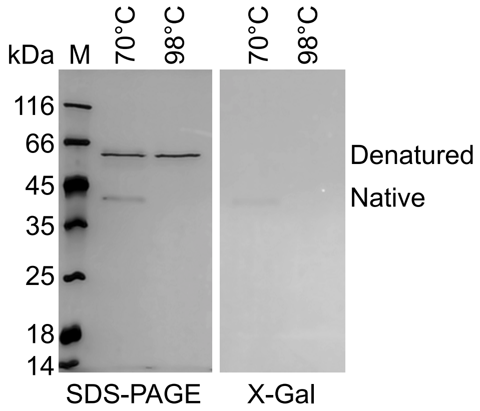

Fig. S3 - SDS-PAGE analysis and zymogram of Bgl1 after incubation at two different temperatures. Purified protein was either incubated at 70°C for 5 min or 98°C for 10 min, respectively. Silver staining indicates the presence of two conformations (completely denatured and native) after incubation at 70°C, while protein is completely denaturated at 98°C. Catalytic activity after refolding of proteins and incubation in 10 mM $NaPO_4$-buffer using X-Gal as substrate is detected only in case of the non-denatured protein form.

A

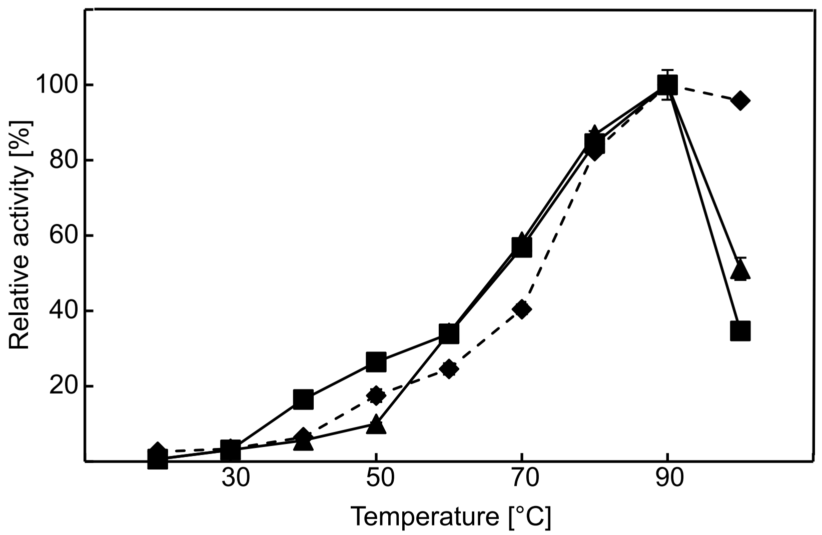

B

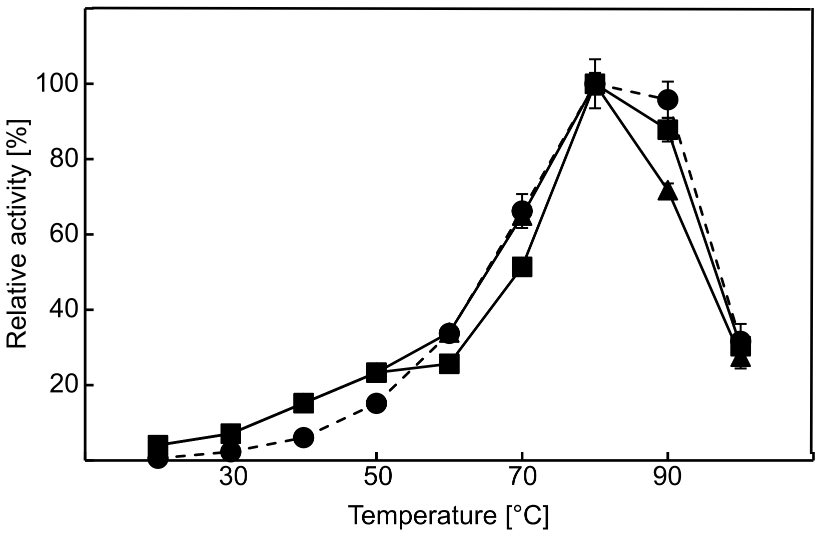

Fig. S4 - Influence of temperature on mutated fusion constructs compared to singular Cel5A and Bgl1. (A) Purified proteins were tested using β-glucan as substrate at different temperatures and constant pH 6. Experiments were done using Cel5A (filled diamonds, dashed line), $C^{E294A}B$ (filled squares, continuous line) and BC^{E294A} (filled triangles, continuous line). (B) Measurement of enzymatic activity towards 4-NP-b-D-GP. Experiments were done using Bgl1 (filled circles, dashed line), CB^{E395G} (filled squares, continuous line) and $B^{E395G}C$ (filled triangles, continuous line). All experiments were done in triplicate.

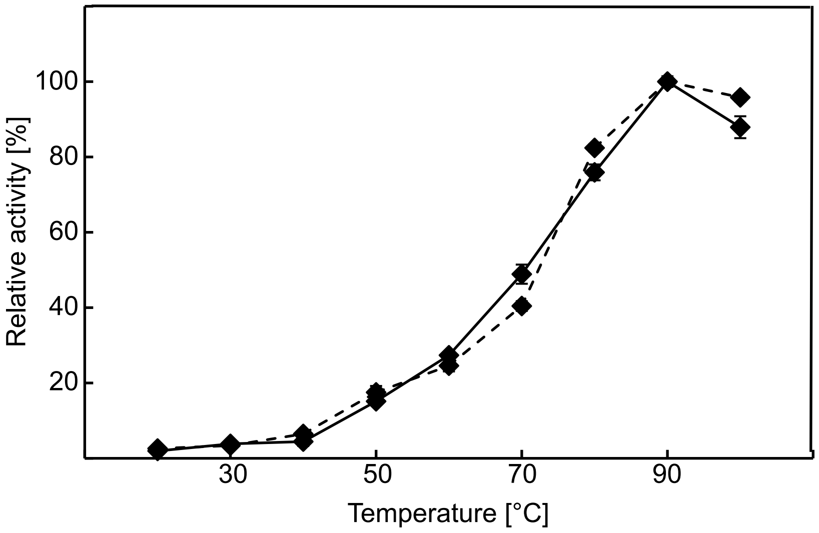

Fig. S5 - Influence of temperature on singular Cel5A and Cel5A in enzyme mixture. (A) Purified proteins were tested using β-glucan as substrate at different temperatures and constant pH 6. Experiments were done using Cel5A alone (filled diamonds, dashed line), and Cel5A in mixture with Bgl1 (filled diamonds, continuous line). All experiments were done in triplicate.

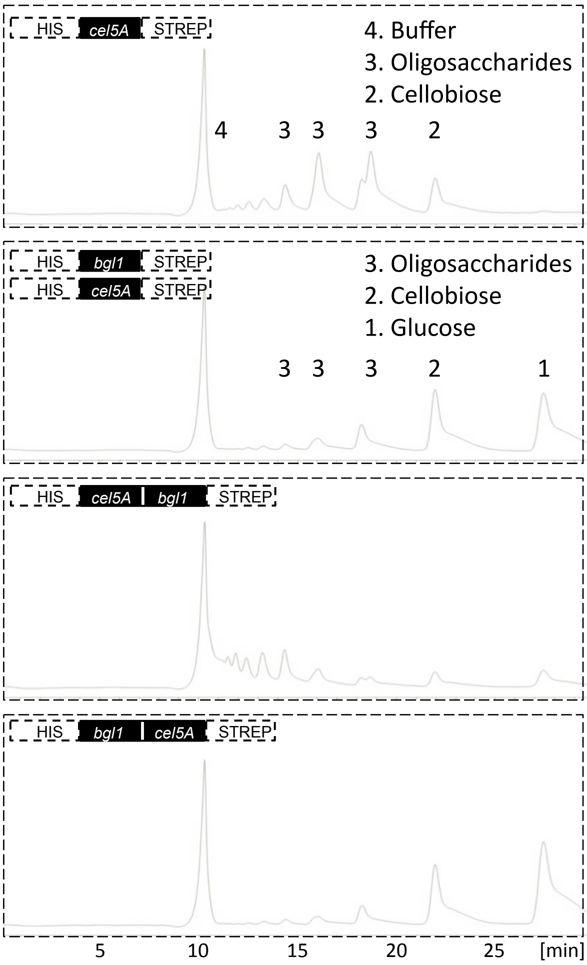

Fig. S6 - HPLC analyses to investigate catalytic activity of fusion enzymes towards cellobiose. The following sugar components were separated under standard conditions using the 1260 Infinity LC system equipped with Hi-Plex Na column and with a R_i-detector from Agilent applied with MilliQ-water used as mobile phase: (1) glucose, (2) cellobiose, (3) different oligosaccharides. Moreover a buffer peak (4) was visible in all measurements. Cellulase was incubated alone (upper panel), 1:1 mixture of endoglucanase and β-glucosidase was incubated (2nd panel), fusion enzymes CB (3rd panel) and BC (4th panel) were used for measurements.

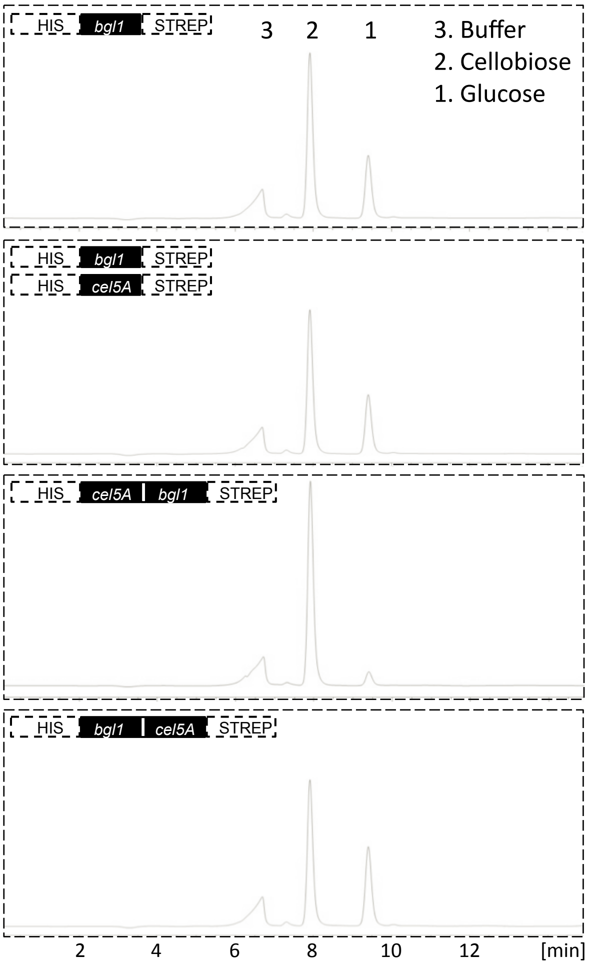

Fig. S7 - HPLC analyses to investigate catalytic activity of fusion enzymes towards β-glucan. The following sugar components were separated under standard conditions using the 1260 Infinity LC system equipped with Hi-Plex H column and with a R_i-detector from Agilent applied with MilliQ-water used as mobile phase: (1) glucose and (2) cellobiose. Moreover a buffer peak (3) was visible in all measurements. β-Glucosidase was incubated alone (upper panel), 1:1 mixture of endoglucanase and β-glucosidase was incubated (2nd panel), fusion enzymes CB (3rd panel) and BC (4th panel) were used for measurements.
